# A New Treatment for Consumption

**Published:** 1897-04

**Authors:** 


### EDITORIAL DEPARTMENT.

F. E. DANIEL, M. D., Editor.
S. E. HUDSON, M. D., Managing Editor.
A. J. SMITH. M. D., Galveston, Associate Editor.

EDITORIAL STAFF:

PROF. J. E. THOMPSON, M. D., Texas Medical College, Galveston; Surgery.
PROF. WM. KEILLER, M. D., Texas Medical College, Galveston; Obstetrics and Gynecology.
PROF. DAVID CERNA, M. D., Texas Medical College, Galveston; Therapeutics.
PROF. A. J. SMITH, M. D., Texas Medical College, Galveston; Medicine.
DR. R. H. L. BIBB, City of Mexico; Foreign Correspondent.

Published Monthly at Austin, Texas, by Drs. Daniel and Hudson. Subscription price $2.00 a year in advance.

Eastern Agents: The Monihan-Fairchild Special Agency, 24 Park Place, New York City.

Official organ of the West Texas Medical Association, the Houston District Medical Association, the Austin District Medical Society, Brazos Valley Medical Association, the Galveston County Medical Society, and several others.

### A NEW TREATMENT FOR CONSUMPTION.

"Is there no balm in Gilead? Are there no physicians there?"

Seeing that the dread disease, consumption, is steadily pursuing its course of devastation, unchecked, despite the vaunted advanced in medicine, the victims may well ask, is there no cure, no hope; are there no physicians equal to the task?"

For years Texas was regarded, especially by the "unco' gude" of the "land of the righteous," as a kind of Botany bay, an out-of-the-way place where people were half civilized and wholly unenlightened; half man, half horse and half alligator; and many are the jokes (?) gotten off at her expense. Sheridan's alleged witticism, for instance, "rent Texas and live in hell" had become a classic. Readers will recall the inquiry of one of our British colleagues "What good can come out of Texas?" As late as twelve years ago, when the TEXAS MEDICAL JOURNAL said; "Let there be light" (and there was light), it was a matter of surprise to some that there were physicians in Texas, sufficiently well educated to publish, or even read a medical journal.

But that is past now, and it is recognized that the physicians of the Lone Star State are the peers of any, anywhere, the palm being, by the medical press, awarded to her State Medical Society for the excellence of her Society Transactions.

Still, it would be a matter of surprise to some,—though it should not be, were Texas yet to furnish a solution to the problem of successfully preventing and treating consumption. Indications presently to be mentioned, point to a strong probability that such may be the case; at least, a Texas physician has made a move in the direction which may lead to that end.

With a knowledge of the etiology and pathology of consumption—germicides have been looked to for the successful treatment; and the desideratum has been to find one that, while harmless to the patient, will be destructive to the pathogenic organisms; and this is the basis of success claimed for the new treatment of typhoid fever by disinfecting the bowel by minute doses of the bichloride. But something more is necessary than the destruction of the germ. In most advanced cases of consumption, the blood,—the whole system is poisoned by the waste product, as well as the product of bacterial action; hence the fever and other symptoms. Creasote will kill the germs—perhaps, but, as said,—something more is necessary; the objection to it being the intolerance of doses sufficiently large or often, to be effective.

The indications to be met in an average case of consumption in the stage of suppuration, when the *bacilli* are seen in the sputa,—and the symptoms indicate a septic condition generally, fever, emaciation, sweats, diarrhœa, are (a) to destroy the *bacilli* in the lung; remove the cause; (b) to counteract or neutralize the poisonous products, i. e., to "disinfect" the system; (c) to eliminate the *debris*—the waste products and the poisonous products of bacterial action; (d) build up the strength and restore wasted tissues.

To meet these indications various remedies have been tried in various ways. A number of persons have cried "Eureka," and straightway turned quack, to profit by their find. Others more honest, have hastened to give the profession the benefit of their observation and experiments, but so far, the cure, like an *ignis fatuus*, has still eluded the grasp, still lures to its pursuit.

Clinical experience has about demonstrated that formaldehyde is the most promising remedy to meet the first indication. It is the surest, yet safest, germicide. It is believed it will yet play an important part in the successful treatment of consumption, and already there are numerous devices on the market for generating it.

Dr. Lewis W. Cock, a Texas physician, residing at San Marcos, a graduate of Tulane (Medical department) University, and a Vanderbilt University alumnus, has, for several years been working on the problem, how to destroy the *baccilli* without harm to the patient; and believing that he was on the right track, spent two years in Chicago where he could have facilities for testing his views. His researches and the result of his experiments have led him to conclude that a union of the gasses of sulphurous acid, formaldehyde, oxygen, and perhaps others, furnish the ideal germicide and disinfectant for use in consumption, and his plan is to generate these vapors, which he does by a device of his own, and under heavy atmospheric pressure force their absorption into a certain volume of water. The chemistry of the product is very complex, and he does not undertake to give its precise formula. He believes this form of administration to be the best, because it is readily taken, is not attended with any danger to the patient, and causes no unpleasant effects; but chiefly because—water—nature's great diluent—being the vehicle—the germ destroying elements are rapidly carried into the circulation and diffused throughout the system, flushing every part; diuresis and peristalsis are stimulated, as well as all glandular action; hence the rapid elimination from the system of all waste products and poisonous matter; the appetite and the digestion are stimulated, and improved nutrition, one of the chief desiderata in the treatment of the disease takes place.

Doctor Cock informs us that from observation in a large number of cases of consumption in which the remedy has been used, all these indications are fully met. At first, the cough, and the amount of expectoration are increased, and under the microscope the sputa is found swarming with the tubercle *bacilli;* after which comes a diminution in the quantity of expectorated matter, an amelioration of all the symptoms, a subsidence of the night sweats, the temperature falls to normal, etc., that at the end of, say three weeks, under the daily administration of the preparation, the *bacilli* disappear from the field under microscopic examination. The physicians of Chicago appear to have become much interested in Dr. Cock's plan, and he informs us that some of them have submitted it to the crucial test of microscopic examination of the sputa, and are enthusiastic in its behalf. Many high testimonials were voluntarily given him.

Knowing our interest in all matters that concerns the legitimate practice, he has taken some pains to make us acquainted with the subject, and has placed at our disposal quite a bulk of such documents.* We have room for only one or two, however, and believing that the doctor's claims are worthy of being presented to our readers, and knowing the doctor as we do, to be an ethical and well read physician, in excellent standing, socially and professionally, we reproduce here the letters of Professor Harper, the chemist of Rush Medical College, and one from Dr. Rufus Bartlett, well and favorably known to the profession.

Professor Harper says:

"CHICAGO, ILL., Dec. 14, 1896.

"MY DEAR DR. COCK:—It affords me pleasure to inform you that after observing the effects of your anti-bacilli compound [for want of a better name?—ED.], and from data given me by a number of my colleagues, that you have discovered a true specific for tuberculosis. Hoping the remedy may receive the merit it richly deserves,

"I am, fraternally yours,

[Signed] "J. E. HARPER, M. D."

(It is well enough to state that Dr. Cock does not claim to have discovered a "specific for tuberculosis," but he says that *so far as observation has extended*, the bacilli have entirely disappeared from the sputa after use of the remedy, as demonstrated by daily microscopic examination, and that all *other symptoms* disappear.)

Dr. Flood, a leading practitioner of Chicago, knowing that Dr. Rufus Bartlett, a well known chemist and practitioner of that city, had been using Cock's preparation, addressed a request to him for information as to results, to which Dr. Bartlett replied as follows:

"CHICAGO, October 15, 1896.

"MY DEAR DR. FLOOD:—Replying to your letter of inquiry in regard to the study and use of Dr. Cock's remedy, will say that it has proved very satisfactory in my hands. Perhaps I can do no better at the present time than to briefly state a few facts and give a few results of treatment without going into the clinical history of cases treated.

---

* The TEXAS MEDICAL JOURNAL as is well know, is intolerant of everything that pertains to quackery, and is on record as always denouncing it without fear or favor. It is hoped that it is equally ready to recognize merit in whatever form it appears, and to give a helping hand to all that is likely to be of value to humanity and to the medical profession. So many "cures for consumption" have been claimed, that we, like others, are suspicious, and on the outlook for the cloven foot.

"I have seen all the distressing symptoms in early consumption slowly and surely disappear, and have noted with wonder the rapid return of strength, and weight, and ambition, where thirty days previous had been elevated temperature, night sweats, debility, and distressing cough, and the *bacilli tuberculosis*, which entirely disappeared under microscopical observation, with the remedy as the only regular medicine. One very interesting case I still have under treatment, is cancer of the stomach, complicated with ulcerations and frequent hemorrhages. The case was rapidly growing worse under all previous treatments, but has steadily gained under this remedy, until all symptoms have given away to increased health, weight and improved appetite. I have also used it in typhoid fever cases with good results, and I myself have found it the surest and speediest relief in *la grippe*. I sincerely hope this will interest you to try this valuable remedy in your practice, and trusting you will give me the benefits of your chemical observations,

"I am, sincerely yours,

"RUFUS BARTLETT, M. D."

This is strong testimony.

Dr. Cock asks that the profession will investigate the matter, and let the microscope be the arbiter of his claim. He solicits correspondence with those having charge of consumptive cases, with a view to testing the remedy. It will be sold only through the medical profession.

If subsequent observation in a larger number of cases should correspond with results already obtained, it may well be said that "good can come out of the Texas Nazareth," and that the Texas Gilead is not without balm nor physicians.

